# Diagnostic and Therapeutic Challenges in a Patient with Synchronous Very High-Risk Prostate Adenocarcinoma and Anal Carcinoma

**DOI:** 10.3390/curroncol29010033

**Published:** 2022-01-15

**Authors:** Jonathan Wallach, Irini Youssef, Andrea Leaf, David Schwartz

**Affiliations:** Veterans Affairs New York Harbor Healthcare System, State University of New York-Downstate Medical Center, New York, NY 11209, USA; Irini.Youssef@Downstate.edu (I.Y.); Andrea.Leaf@va.gov (A.L.); David.Schwartz3@va.gov (D.S.)

**Keywords:** prostate adenocarcinoma, anal carcinoma, synchronous cancers

## Abstract

A 79-year-old HIV-negative Caucasian man with a medical history of smoking 20 pack-years (quit 40 years prior), early-stage non-small cell lung cancer status post-lobectomy 13 years earlier at an outside hospital without evidence of recurrence, and benign prostatic hypertrophy was diagnosed with synchronous very high-risk prostate adenocarcinoma and early-stage anal basaloid squamous cell carcinoma. He proceeded to undergo concurrent treatment for these tumors, consisting of androgen deprivation therapy, external beam radiation therapy, and a brachytherapy boost for the prostate adenocarcinoma; for the anal carcinoma, he was treated with definitive chemoradiation. Over 3.5 years since the completion of radiotherapy, he remains in clinical and biochemical remission.

## 1. Introduction

### Case Description

A 79-year-old HIV-negative Caucasian man with a medical history of smoking 20 pack-years (quit 40 years prior), early-stage non-small cell lung cancer (subtype unknown) status post-lobectomy 13 years earlier at an outside hospital without evidence of recurrence, and benign prostatic hypertrophy began demonstrating accelerating prostate specific antigen (PSA) values, prompting a prostate biopsy by his urologist. His previously stable PSA had increased from 4.3 ng/mL to 6.1 ng/mL three months later, confirmed by repeat laboratory drawing. On physical examination, his prostate was estimated as 45 g and was smooth, without any nodularity appreciated; however, there was also a course lesion along the posterior anal canal. He proceeded with a 12-core ultrasound-guided biopsy, which demonstrated 7/12 cores with tumor, including one core with Gleason 5 + 5 = 10 (grade group 5), one core with Gleason 4 + 5 = 9 (grade group 5), one core with Gleason 5 + 3 = 8 (grade group 4), one core with Gleason 4 + 3 = 7 (grade group 3), and three cores with Gleason 3 + 3 = 6 (grade group 1); there was no perineural invasion or lymphovascular invasion.

A flexible sigmoidoscopy revealed that the anal lesion measured 2 cm and was the only lesion of concern. A biopsy of the anal mass demonstrated grade 3 basaloid squamous cell carcinoma, positive for human papilloma virus (HPV) subtypes 16/18 and negative for HPV subtypes 6/11 and 31/33.

He underwent a bone scan, demonstrating a non-specific focus within the left lateral 10th rib, representing a healed fracture versus a metastasis. A CT-chest/abdomen/pelvis with contrast did not demonstrate any lymphadenopathy or osseous lesions; his bladder measured 5.9 × 5.4 cm, indenting the bladder base; the anal lesion was poorly visualized. He underwent a PET/CT, demonstrating normal uptake within the lungs, a significantly enlarged prostate, no lymphadenopathy, no metabolically active lesions within the bones, and focal intense activity within the anal region (consistent with the recent biopsy). An MRI-prostate was deferred at that time because of concern for potential incompatibility of a prosthesis with the magnetic field. Therefore, his diagnosis was synchronous very high-risk prostate adenocarcinoma cT1cN0M0 (grade group 5, pre-treatment PSA = 6.1 ng/mL, stage IIIC) concurrent with cT1N0M0 stage I anal basaloid squamous cell carcinoma, per the AJCC 8th Edition staging system.

**Figure 1:**
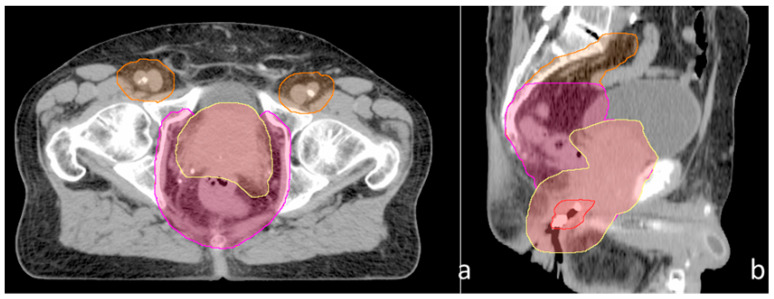
(**a**) Axial view, (**b**) sagittal view.

The patient’s case was discussed at a multidisciplinary tumor board. Per the recommendations, he was started on bicalutamide 50 mg daily, followed by leuprolide acetate 22.5 mg every 3 months after 3 weeks on bicalutamide. The patient underwent prophylactic radiotherapy to his breast buds, consisting of 12 Gy over 4 fractions with 6 MeV en face electrons to prevent gynecomastia. The patient started volumetric modulated arc therapy (VMAT) to the prostate, seminal vesicles, anus, pelvic lymph nodes, and inguinal lymph nodes, as seen in Figure 1, at the radiotherapy doses described in Table 1; radiotherapy was concurrent with two cycles of 5-fluorouracil 1000 mg/m^2^/day on days 1–4 and 29–32 and mitomycin-C 10 mg/m^2^ on days 1 and 29.

The patient tolerated treatment with some difficulties, as he did experience CTCAE grade 3 dehydration (requiring inpatient intravenous hydration), grade 2 neutropenia treated with filgrastim, grade 2 diarrhea treated with loperamide 2 mg 3x/day PRN, grade 2 urinary frequency treated with tamsulosin 0.4 mg qhs, and grade 1 fatigue. From consultation to the end of this part of treatment, he lost 4.2 kg, which was 5.0% of his initial weight (grade 1 weight loss), while his International Prostate Symptom Score (IPSS) increased from 7 to 11 on tamsulosin 0.4 mg.

He remained on bicalutamide 50 mg daily and leuprolide acetate 22.5 mg q3 months and underwent planning for prostate brachytherapy. Two months later, he returned for low dose rate (LDR) brachytherapy with hydrogel placement between the prostate and the rectum. The I-125 implant consisted of 60 seeds and delivered a 108 Gy boost. He tolerated the procedures well without any complications; the dosimetry was as follows: prostate D90 = 118.88 Gy, prostate V100 = 97.85%, rectum V100 = 0.00 cc, and urethra V150 = 0.00 cc.

Following the brachytherapy implant, the patient continued to receive leuprolide acetate and bicalutamide for a total duration of three years, given his very high-risk disease. He has continued to follow up with urology, radiation oncology, medical oncology, pulmonology, and the surgical service, given his history of three malignancies and emphysema.

With regard to his surveillance, the patient most recently followed up with the surgical service at 3 years and 3 months after the completion of radiotherapy, with no evidence of any anal tumor on physical examination. An MRI-prostate was performed at 2 years 10 months after his radiotherapy, ordered by the surgical service because of painless rectal bleeding for 1 day; the MRI demonstrated no evidence of prostate malignancy, anal malignancy, lymphadenopathy, or osseous lesions. PSAs performed at 2 years 10 months, 3 years 5 months, and 3 months 8 months were 0.02 ng/mL, 0.04 ng/mL, and 0.05 ng/mL, respectively, demonstrating biochemical control. 

Of note, the patient did experience increased lower urinary tract symptoms that required botulinum injection twice during post-radiotherapy year 3, and he briefly had a urinary catheter. However, his last IPSS evaluation at 3 years 8 months post-radiotherapy demonstrated an IPSS score of 0/1/4/0/5/3/1 = 14 on tamsulosin 0.4 mg × 2 daily. In addition, during post-radiotherapy year 2, he experienced minor rectal bleeding, which self-resolved.

Additionally, a PET/CT at 3 years 5 months post-radiotherapy demonstrated no evidence of the prostate or anal malignancy but did reveal a new PET-avid pleural-based nodule at the right hemi-diaphragm. A right lower lobe core biopsy demonstrated poorly differentiated lung adenocarcinoma, consistent with a new lung primary. He proceeded with stereotactic body radiation therapy to this focus consisting of 50 Gy over 4 fractions, which he tolerated very well without any acute toxicities.

## 2. Discussion 

Anal cancer is a relatively rare malignancy in the United States, accounting for approximately 2% of all gastrointestinal malignancies [1,2], and is estimated to have been diagnosed in 8590 Americans in 2020 [3]. However, its incidence is increasing because of two major factors: longer survival of patients infected with HIV as a result of successful use of HAART, as well as an increase in the rate of sexually transmitted HPV [4]. Generally, the management of anal cancer includes concurrent chemoradiation (CCRT), with surgery reserved for persistent or recurrent disease [2]. 

Prostate adenocarcinoma is the most common non-skin cancer in American men, with about 1 in 9 men diagnosed with prostate cancer during their lifetimes [5]. The management paradigm for prostate cancer differs by National Comprehensive Cancer Network (NCCN) risk group, with radical prostatectomy and radiotherapy as the main treatment options, while men with very low-risk, low-risk, and potentially favorable intermediate-risk prostate cancer can pursue active surveillance [6]. For men with at least unfavorable intermediate-risk prostate adenocarcinoma, androgen deprivation therapy is generally added to radiotherapy after taking into consideration co-morbidities and side effects [6].

Additionally, when treating these more aggressive tumors with radiotherapy, the NCCN recommends the consideration of a brachytherapy boost [6]. The ASCENDE-RT trial demonstrated that compared with dose-escalated EBRT to 78 Gy, men who received a boost with LDR brachytherapy were twice as likely to be free of biochemical failure at a median follow-up of 6.5 years [7]. However, despite this category A evidence, as well as numerous prospective and retrospective studies showing improved outcomes with a brachytherapy boost, an analysis of the National Cancer Database from the years 2004–2012 reported a significant decline in the use of prostate brachytherapy at both academic and non-academic institutions, from 15% to 8%, and from 19% to 11%, respectively [8]. For this patient, we chose an LDR brachytherapy boost (instead of a high dose rate [HDR] boost) to be consistent with the ASCENDE-RT trial [7]. The use of brachytherapy with the hydrogel spacer allowed delivery of a boost dose to the prostate while minimizing the dose to the anus after the completion of anal CCRT. 

Because of the rarity of synchronous presentations of anal and prostate malignancies, there is almost no available literature on an optimal simultaneous treatment strategy. In one case report by Miles et al. of a 68-year-old man with synchronous unfavorable intermediate-risk prostate and anal cancers, the authors initially utilized a 3D-conformal approach to treat the primary diseases and draining lymph nodes with conedown fields to the anus to a dose of 50.4 Gy, followed by an IMRT boost to 73.8 Gy to the prostate and proximal seminal vesicles, with neoadjuvant and concurrent androgen deprivation therapy [1]. Another case report by Tubin et al. demonstrated the use of definitive IMRT with a simultaneous integrated boost in the management of a 68-year-old Caucasian male with synchronous anal cancer with nodal metastases and intermediate-risk prostate cancer through the use of four planning-treatment volumes: the uninvolved inguinal lymph nodes up to 36 Gy, uninvolved pelvic lymph nodes up to 45 Gy, involved pelvic lymph nodes and primary anal tumor up to 59.4 Gy, and the prostate up to 69.3 Gy [9]. The inguinal lymph nodes were included as a low-dose region, despite being negative on imaging, per the landmark protocol RTOG 05–29 [10].

An important decision for patients diagnosed with synchronous malignancies is whether both tumors require definitive management. Given that our patient had very high-risk prostate cancer, active surveillance was not an option, as men in this risk group have poor outcomes even with very aggressive treatment, with a cancer-specific survival of 62% at 10 years [11]. Additionally, given his age and comorbidities, he was not medically optimal for surgical resection. 

Our approach to the management of this patient combined elements from the standard of care for both malignancies. It was necessary to dose the prostate to at least 78 Gy on the basis of randomized trials demonstrating prostate cancer mortality benefits [12] as well as lower biochemical failure and distant metastatic rates [13]. The doses used to treat anal cancer are thus insufficient for prostate cancer control. Additionally, taking both the anus and prostate to such high doses would result in significant anal toxicity. In order to overcome this issue, we delivered a maximum dose of 50.4 Gy to the prostate, seminal vesicles, and anus/perianal regions, with concurrent mitomycin/5-fluorouracil as the standard of care; we added a brachytherapy boost to the prostate to a dose of 108 Gy using the I-125 isotope. This modality allowed treatment of both malignancies to the recommended doses while sparing normal tissue. 

Additionally, based on this patient’s risk of lymph node involvement of 31% using the MSKCC nomogram [14], we decided to include the pelvic lymph nodes in the treatment volume, regardless of whether the patient had a synchronous anal malignancy. Fortunately, this allowed us to combine pelvic volumes for both the prostate and anal carcinomas. Finally, we incorporated androgen deprivation therapy into his management strategy on the basis of several randomized trials showing a benefit to 2–3 years of androgen deprivation in combination with radiation for high-risk patients [15,16,17]. With this dual treatment approach, the patient was able to tolerate treatment well, with manageable acute toxicity and long-term toxicity. 

## 3. Conclusions

In summary, there is almost no medical literature on the optimal treatment strategy for patients with synchronous prostate and anal cancers, especially because of the rarity of anal cancer. After it was determined that each malignancy warranted definitive management, we determined that a combined radiation approach, especially one utilizing brachytherapy because of its rapid dose fall-off, in addition to sensitizing chemotherapy, was the most effective strategy. Our experience suggests that this was a well-tolerated and effective strategy for this patient and can be replicated in similar clinical scenarios. Additionally, this case report of a patient with four synchronous/metachronous tumors in three separate organs reinforces the premise that a patient who develops a malignancy is at an elevated risk for developing additional malignancies, both because of systemic genetic derangements and environmental exposures; consideration of genetic counseling and behavior modifications (e.g., tobacco and alcohol cessation) should be considered in appropriate circumstances.

## Figures and Tables

**Table 1:** Treatment Schema.

Structure	Region 1	Region 2	Region 3	Fractionation	Figure 1
PTV 36	Common iliac LNs, inguinal LNs	External iliac LNs, internal iliac LNs, obturator LNs	Prostate, seminal vesicles, anus/peri-anal region	36 Gy/18 fractions	Orange
PTV 45		External iliac LNs, internal iliac LNs, obturator LNs	Prostate, seminal vesicles, anus/peri-anal region	45 Gy/25 fractions	Magenta
PTV 50.4			Prostate, seminal vesicles, anus/peri-anal region	50.4 Gy/28 fractions	Yellow (anus in red)

## Data Availability

The data presented in this study are available on request from the corresponding author.
